# An Alarming Mimicry of Intra-Abdominal Infections: Acute Appendiceal Diverticulitis

**DOI:** 10.1155/2021/6131015

**Published:** 2021-11-12

**Authors:** Youseung Kim, Varun Kesar, Douglas Grider, Maithili V. Chitnavis

**Affiliations:** ^1^Virginia Tech Carilion School of Medicine, Department of Internal Medicine, Roanoke, VA, USA; ^2^Virginia Tech Carilion School of Medicine, Department of Internal Medicine, Division of Gastroenterology and Hepatology, Roanoke, VA, USA; ^3^Virginia Tech Carilion School of Medicine, Department of Basic Science Education, Roanoke, VA, USA; ^4^Dominion Pathology Associates, Roanoke, VA, USA

## Abstract

A 65-year-old woman presented with three days of colicky abdominal pain. Abdominal imaging illustrated small bowel enteritis, ascites in both paracolic gutters, and incidental hepatic steatosis. Although ascites fluid demonstrated high neutrophil count consistent with peritonitis and the patient received adequate antibiotics, she clinically deteriorated. Subsequent exploratory laparotomy revealed necrotic appendix and multiple intra-abdominal abscesses. Histopathology showed acute suppurative appendicitis with multiple other intact small diverticula, indicating likely perforation of inflamed appendiceal diverticula with subsequent abscess formation and abdominal peritonitis. This case highlights the importance of ascites fluid analysis and continued clinical correlation, especially in cases of rare entities with atypical presentations.

## 1. Introduction

Diverticula are small protrusions formed from the walls of the tubular gastrointestinal tract and can be associated with several complications such as hemorrhage, inflammation, and perforation. Anatomically, there are two types of diverticula—true and false. True diverticula involve all three layers of the intestinal wall, which consists of the mucosa, submucosa, and muscularis propria, and are most recognized in congenital diverticula such as Meckel's diverticula. False diverticula consist of only mucosa and submucosa without the involvement of the muscularis propria and are associated with acquired diverticulosis. Not surprisingly, many colonic diverticula are false diverticula and originate from weak points in the intestinal wall [1]. Acute appendiceal diverticulitis is a rare pathology of the appendix with the incidence of 0.004–2.1% [2] and is described in the literature by isolated case reports [3, 4]. We present a case of acute appendiceal diverticulitis with delayed diagnosis complicated by perforation of the diverticulum, abscess formation, and peritonitis.

## 2. Case Presentation

**Figure 1:**
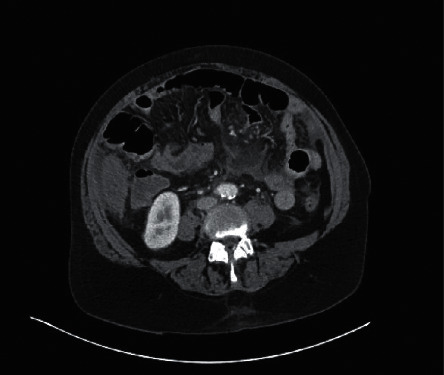
Initial computed tomography (CT) of the abdomen and pelvis with small bowel enteritis and fluid collections in paracolic gutters.

A 65-year-old woman with a history of essential hypertension presented with three days of intermittent, left lower quadrant colicky abdominal pain and nonbilious vomiting, a few hours after an outdoor barbecue, along with subjective fever, chills, and dizziness. Abdominal and pelvic computed tomography (CT) with contrast showed signs of small bowel enteritis (Figure 1). Incidental findings included diffuse hepatic steatosis and small amounts of ascites in both paracolic gutters.

**Figure 2:**
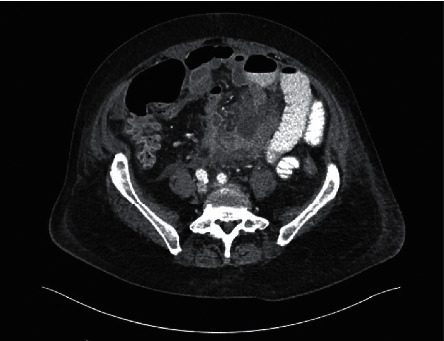
Repeat computed tomography (CT) of the abdomen and pelvis with new peritoneal enhancement suggestive of peritonitis.

At admission, the emergency general surgery service was consulted and recommended intravenous hydration and treatment for infectious enteritis under the general medicine service. A diagnostic paracentesis on the second day of hospitalization revealed an ascitic fluid white blood cell (WBC) count of 18,800 cells/mm^3^ with 75% neutrophils, 12% lymphocytes, 13% mononuclear cell, glucose of 94 mg/dL, protein of 3.9 g/dL, and LDH of 459 IU/L. The patient was continued on cefepime for possible spontaneous bacterial peritonitis (SBP). However, by the third day of admission, the patient's abdominal pain had intensified. The gastroenterology team was consulted for the management of ascites. On examination, the patient had a distended, rigid abdomen with voluntary abdominal guarding and diffuse significant tenderness to palpation suggestive of peritonitis. Given that diagnostic paracentesis was also suspicious for secondary bacterial peritonitis by Runyon's criteria [5], a repeat CT of the abdomen and pelvis with contrast and surgical reconsultation were recommended. CT illustrated worsening moderate ascites without free air, but with new peritoneal enhancement, suggestive of peritonitis (Figure 2), along with the concern for small bowel obstruction (SBO) in the left lower abdominal quadrant.

**Figure 3:**
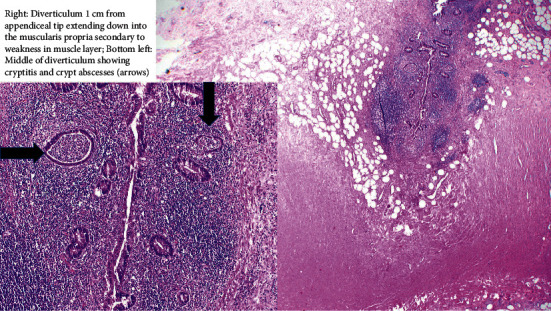
Histopathology of appendiceal diverticulitis in the portion of the remaining viable appendix.

The patient was initially treated conservatively for a possible SBO, but due to continued worsening abdominal pain, she subsequently underwent diagnostic laparoscopy. This revealed multiple pockets of pus and purulent peritonitis. Consequently, the laparoscopy was converted to open laparotomy with drainage of intraloop, right lower quadrant, and pelvic abscesses. Appendectomy for a necrotic-appearing appendix was also performed. Histopathology showed acute suppurative appendicitis and periappendicitis with multiple small diverticula along the length of the remaining viable appendix, supporting the likely perforation of one or more diverticula secondary to acute inflammation with subsequent abscess formation and abdominal peritonitis (Figure 3). The patient completed a total of ten days of antibiotics and was discharged with clinical improvement.

## 3. Discussion

There are two classifications of diverticula: congenital or acquired. Congenital diverticulum, or true diverticulum, is a rare entity, with minimal incidence of perforation because it includes all the layers of mucosa, submucosa, and muscularis propria. By comparison, an acquired diverticulum is a false diverticulum that does not involve the muscularis propria and therefore has higher tendency for perforation [6, 7].

In retrospect, this patient presented with nonspecific symptoms of appendiceal diverticulitis of false diverticula. The patient's nonspecific gastrointestinal symptoms including abdominal pain and ascites along with initial imaging concerning for parenchymal liver disease led to a suspicion for SBP. Despite antibiotic therapy, the patient continued to worsen with peritoneal signs on abdominal exam and persistent leukocytosis. Moreover, elevated ascitic total protein and LDH were highly concerning for secondary peritonitis by fulfilling two of three Runyon's criteria, which has a sensitivity and specificity of 67 and 96%, respectively [5]. This prompted repeat imaging and subsequent laparotomy revealing purulent peritonitis, the likely source being perforated suppurative appendiceal diverticulitis.

Delay in the diagnosis and treatment of appendiceal diverticulitis is not uncommon. Appendiceal diverticulitis is often difficult to distinguish from other intra-abdominal infections of the right-sided colon such as infectious ileocecitis caused by *Yersinia*, *Campylobacter*, and *Salmonella* and pseudomembranous colitis caused by *Clostridium difficile* and especially from acute appendicitis, both clinically and radiologically [8]. Clinically, patients with either acute appendicitis or appendiceal diverticulitis will typically present with nonspecific gastrointestinal symptoms, including abdominal pain, nausea, vomiting, and diarrhea. Radiographically, both disease processes illustrate inflammation of the appendix on contrast-enhanced CT studies, though the rate of incidence may differ between them [9]. A retrospective cohort study conducted by Ito et al. reported only 24% visualization of appendiceal diverticulum and 16% of fluid collection in the appendiceal lumen in patients with a pathologically proven diagnosis of appendiceal diverticulitis compared to 100% visualization of the appendix with 88% of fluid collection in the appendiceal lumen in patients with acute appendicitis [9]. Furthermore, the appendix could not be visualized in the CT findings of 48% of the patients in the appendiceal diverticulitis group [9]. Nonspecific clinical and radiological findings can lead to delay in diagnosis as well as treatment of appendiceal diverticulitis, thus increasing the rate of severe complications such as perforation compared to acute appendicitis (65.8% vs. 10.2%) [10].

Recent studies suggest that there may be subtle characteristics in patient presentation that help differentiate between appendiceal diverticulitis and acute appendicitis. For example, appendiceal diverticulitis more commonly presents in older patients compared to acute appendicitis, with an average of 43 versus 29 years, respectively [4]. Also, patients with appendiceal diverticulitis have a longer average duration of time before emergency department presentation compared to that of acute appendicitis, 71 versus 37 hours [11]. These factors lead to an increased rate of severe complications associated with appendiceal diverticulitis, such as perforation, compared to acute appendicitis [10]. Although appendiceal diverticulitis is a rarer entity as compared to acute appendicitis, further characterization and consideration of this entity may benefit to decrease complications and mortality associated with this condition.
